# Update to “guidelines on COVID-19 vaccination in patients with immune-mediated rheumatic diseases: a Brazilian Society of Rheumatology task force”

**DOI:** 10.1186/s42358-022-00256-1

**Published:** 2022-07-28

**Authors:** Anna Carolina Faria Moreira Gomes Tavares, Ana Karla Guedes de Melo, Vítor Alves Cruz, Lilian David de Azevedo Valadares, Ricardo Machado Xavier, Viviane Angelina de Souza, Gecilmara Cristina Salviato Pileggi

**Affiliations:** 1 Clinical Hospital, Federal University of Minas Gerais, Belo Horizonte, Brazil; 2 Lauro Wanderley Universitary Hospital, Federal University of Paraíba, R. Tab. Stanislau Eloy, 585 - Castelo Branco, João Pessoa, Paraíba 58050-585 Brazil; 3 Clinical Hospital, Federal University of Goiás, Goiânia, Brazil; 4 Getúlio Vargas Hospital, Recife, Brazil; 5 Clinical Hospital, Federal University of Rio Grande Do Sul, Porto Alegre, Brazil; 6 Federal University, Juiz de Fora, Brazil; 7 Federal University of São Paulo, São Paulo, Brazil

The Committee of Endemic and Infectious Diseases and the Executive Board of the Brazilian Society of Rheumatology (SBR) proposed an update to the “Guidelines on COVID-19 vaccination in patients with immune-mediated rheumatic diseases: a Brazilian Society of Rheumatology task force” [1] based on recently published scientific evidence [2] and on the new recommendations of the Brazilian National Immunization Program (NIP) for vaccination of immunocompromised persons [3, 4]. According to the NIP documents [3, 4], the primary vaccination series is composed of three doses of Coronavac or ChAdOx-1 (AstraZeneca) or mRNA BNT162b2 (Pfizer), or two doses of Ad26.COV2.S (Janssen). A booster is recommended four months after the primary vaccination schedule, preferably with an mRNA vaccine or a non-replicating viral vector. The NIP recommendations for immunocompromised patients, endorsed by the SBR, are summarized below:

1. An 8-week interval between the second and third doses was chosen over a shorter interval of 3 weeks to potentiate vaccine responses and minimize possible adverse events [3];
2. Persons 18 years and older who received the three-dose primary vaccination series should receive a fourth booster dose four months after the third, preferably with AstraZeneca, Janssen or Pfizer [3];
3. Persons 18 years and older who received one dose Janssen vaccine should complete the primary vaccination series with a second dose of the Janssen vaccine eight weeks after the first dose and receive a third booster dose four months after the second dose, preferably AstraZeneca, Janssen, or Pfizer [3];
4. Pregnant and postpartum women (up to 45 days following end of pregnancy) should receive a booster dose four months after the primary vaccination series of Pfizer vaccine and, if not available, Coronavac vaccine [3];
5. Adolescents from 12 to 17 years old should receive three doses in the primary vaccination series and a fourth booster dose Pfizer four months after the third one [3];
6. Children from 5 to 11 years old should receive the Pfizer two-dose primary vaccination series with an 8-week interval between the first and second doses [4].

## Data Availability

Not applicable.

## Abbreviations

SBR: Brazilian Society of Rheumatology
NIP: National Immunization Program
